# Investigation of span-chordwise bending anisotropy of honeybee forewings

**DOI:** 10.1242/bio.022541

**Published:** 2017-04-10

**Authors:** JianGuo Ning, Yun Ma, HuiLan Ren, PengFei Zhang

**Affiliations:** State Key Laboratory of Explosion Science and Technology, Beijing Institute of Technology, Beijing 100081, China

**Keywords:** Honeybee forewing, Bending stiffness, Resilin, Corrugation, Venation

## Abstract

In this study, the spanwise and chordwise bending stiffness *EI* of honeybee forewings were measured by a cantilevered bending test. The test results indicate that the spanwise *EI* of the forewing is two orders of magnitude larger than the chordwise *EI*. Three structural aspects result in this span-chordwise bending anisotropy: the distribution of resilin patches, the corrugation along the span and the leading edge vein of the venation. It was found that flexion lines formed by resilin patches revealed through fluorescence microscopy promoted the chordwise bending of the forewing during flapping flight. Furthermore, the corrugation of the wing and leading edge veins of the venation, revealed by micro-computed tomography, determines the relatively greater spanwise *EI* of the forewing. The span-chordwise anisotropy exerts positive structural and aerodynamic influences on the wing. In summary, this study potentially assists researchers in understanding the bending characteristics of insect wings and might be an important reference for the design and manufacture of bio-inspired wings for flapping micro aerial vehicles.

## Abstract (precis)

**Summary:** The span-chordwise bending anisotropy is due to the distribution of resilin patches, the corrugation along the span and the leading edge vein of the venation.

## INTRODUCTION

During flight, flapping insect wings undergo dramatic deformations such as significant bending and twisting (Dalton, 1975; Wootton, 1990), which are mainly controlled by the wing architecture and control of the wing base (Ennos, 1988a,b). In a previous study based on the stress relaxation test of a dragonfly wing (*in vitro*), Bao et al. (2006) established a viscoelastic constitutive relation model, revealing that the viscoelastic constitutive relationship more rationally characterizes the material properties of insect wings as opposed to the elastic relationships. Ganguli et al. (2010) point out that the stiffness of a *Calliphora* wing is higher in the basal or root region of the wing and falls dramatically towards the wing tip; at the same time, the wing is stiffer when bending up compared to when bending down, especially near the basal region. This is consistent with the discovery of Lehmann et al. (2011) on the variation of local flexural stiffness along the span of *Calliphora* wings. Mengesha et al. (2011) present a comprehensive experimental analysis of the change in mass and stiffness of gradually desiccating forewings of painted lady butterflies (*Vanessa cardui*), demonstrating the declining speed of wing mass, increasing speed of wing stiffness, and final steady-state levels of wing mass and stiffness.

The spanwise flexibility could possibly increase aerodynamic forces through creating higher effective angles of attack via spanwise deformation (Shyy et al., 2010); whereas the chordwise flexibility can achieve the redistribution of lift versus thrust by changing the projection angle of the wing with respect to the freestream by changing camber deformation (Chimakurthi et al., 2009). Even though several researchers (Ganguli et al., 2010; Mengesha et al., 2011; Combes and Daniel, 2003) have investigated bending properties of insect wing materials, these previous studies are not exhaustive or thoroughly convincing. Therefore, the inherent causes of the bending features of insect wings still require investigation.

In this study, we measured the bending stiffness of honeybee forewings using a cantilevered beam approach, in order to better understand the factors causing the span-chordwise bending anisotropy, through fluorescence microscopy (FM) and high-resolution micro-computed tomography (micro-CT). The honeybee was chosen as the research subject because of its flight kinematics (Altshuler et al., 2005), its known flight capabilities (Mountcastle and Combes, 2013), and its worldwide importance as a pollinator (Wood et al., 2015). In this paper, FM was used to illustrate the influence of resilin distribution on the chordwise bending. Micro-CT (Jongerius and Lentink, 2010) was used to create three-dimensional (3D) high-resolution rendering of the cross-sectional corrugation on the chordwise profile of the forewing, and to aid understanding of the influence of cross-sectional corrugations on the spanwise bending of the forewing. Then, by combining the honeybee forewing venation and previous studies (Combes and Daniel, 2003; Rajabi and Darvizeh, 2013; Ren et al., 2012; Chen et al., 2013; Kesel et al., 1998) on the wing venation, it was found that the leading edge vein was another factor influencing the span-chordwise anisotropy. In summary, even though the span-chordwise anisotropy of insect wings was previously reported, we submit that the published information is incomplete and there is a need, based on our present work, to integrate all the possible factors to explain and discuss this feature as comprehensively as possible.

## RESULTS AND DISCUSSION

### Bending test

**Fig. 1.**
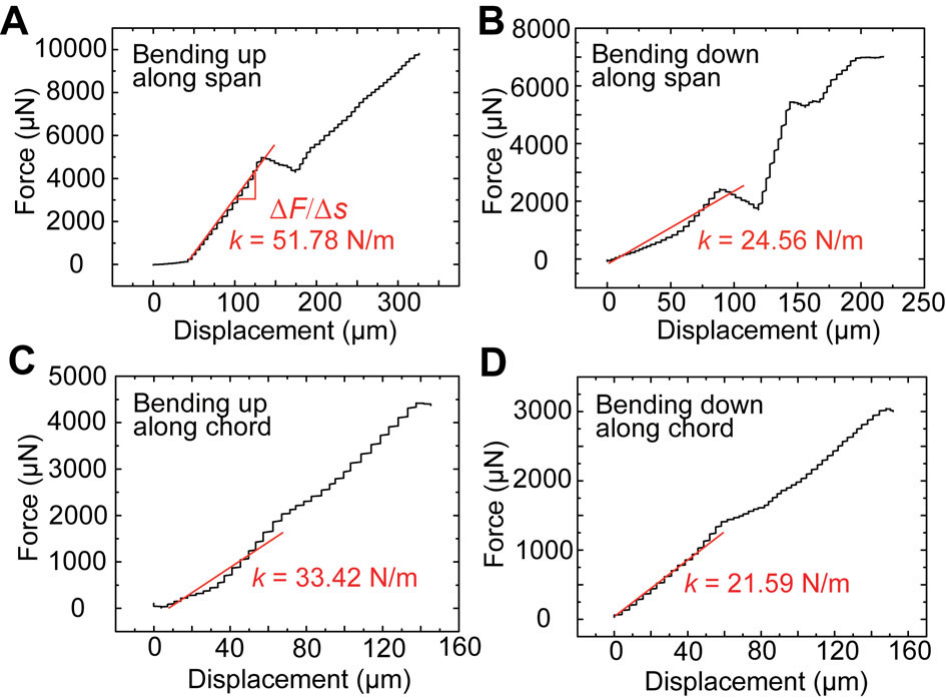
**Force-displacement curves of the spanwise and chordwise bending of the forewing.** The curves of spanwise bending loaded ventrally and dorsally are shown respectively in (A) and (B), and the curves of chordwise bending loaded ventrally and dorsally are shown respectively in (C) and (D).

**Fig. 2.**
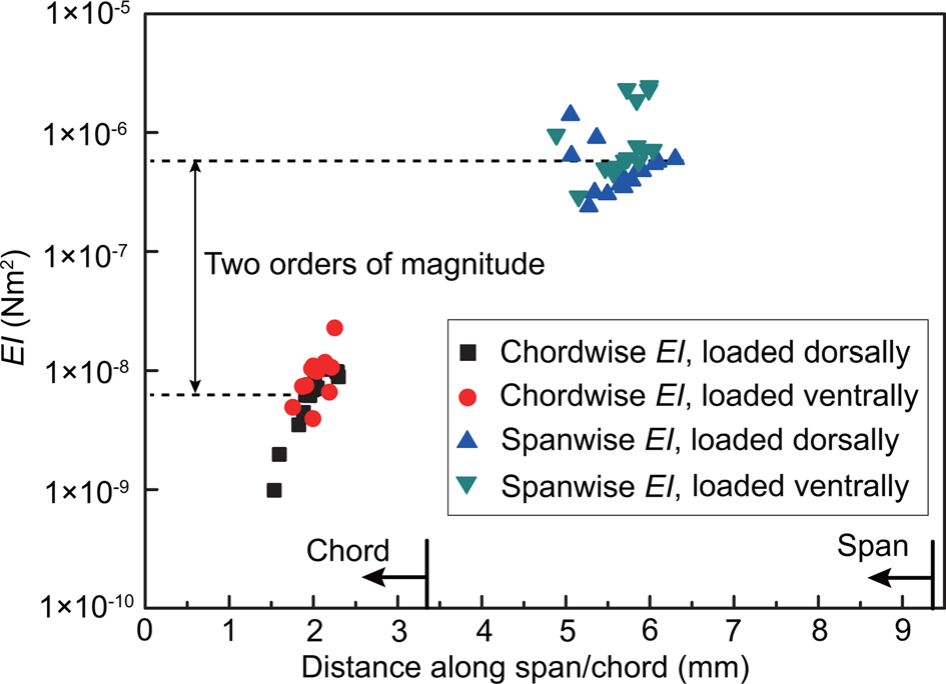
**The spanwise and chordwise bending stiffness *EI* of the forewing.** The vertical axis is on a logarithmic scale. The span and chord length of the forewing are 9.35 mm and 3.31 mm, respectively.

By comparing with the length reference provided by the coin thickness (one jiao, Chinese coin, version 2006) which is 1.67 mm, the effective length *x_F_* (see ‘Bending test’ in Materials and methods) of bending in each test was obtained by calculating the number of pixels of the effective bending length in the captured images which were photographed using Canon EOS 550D (Canon Inc., Japan). There were a total of four types of bending tests performed, i.e. spanwise bending up and down, and chordwise bending up and down. The maximum displacement of the curve is nearly 5% of the effective length of the bending. The slope *k* (10.09–51.78 N/m) of the initial part of the bending test curves (Fig. 1), namely force per unit displacement (Eqn 1), are shown in Table 1. Ganguli et al. (2010) measured the bending stiffness of the wing base, wing centre, and wing tip of *Calliphora* to be from 0.457 N/m to 64.305 N/m, which indicates that the force per unit displacement was also used to represent the bending stiffness. In this study, by including the measurement of the effective bending length *x_F_*, the bending stiffness *EI* of the forewing could be calculated by Eqn 2:
(1)
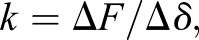
where *k* is the equivalent slope of the force-displacement curve, Δ*F* is the increment of force, and Δ*δ* is the displacement increment of the loading position. The *EI* was calculated over the distance used by Gordon (1978) in the manner used by Combes and Daniel (2003):
(2)
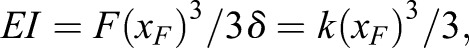
where *F* is the force applied to the wing by a pin and *δ* is the displacement of the loading position. This equation provides a measure of the bending stiffness over the entire wing length. The result shows that the spanwise *EI* of the forewing is two orders of magnitude greater than the chordwise *EI* (Fig. 2), revealing a distinct anisotropy of spanwise and chordwise bending of the honeybee forewing. We find that some variation in the tested *EI* values (Fig. 2) is caused by variability in the specimens, and several data points deviate from the main tendency of the results. However, this variation in the results does not affect the overall quantitative relation between the spanwise and chordwise *EI*.

**Table 1.**
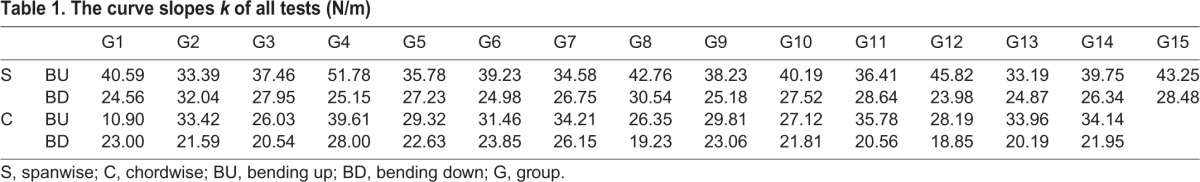
**The curve slopes *k* of all tests (N/m)**

### Fluorescence microscopy

**Fig. 3.**
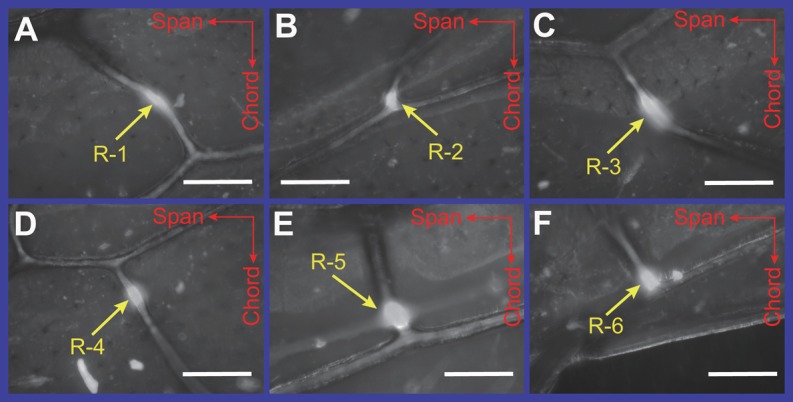
**FM figures of six resilin patches captured from the ventral side of the forewing.** The red horizontal arrows point from the wing root to the wing tip, and the red vertical arrows point from the leading edge of the wing to the trailing edge. R indicates the resilin patch. Scale bars: 100 μm.

**Fig. 4.**
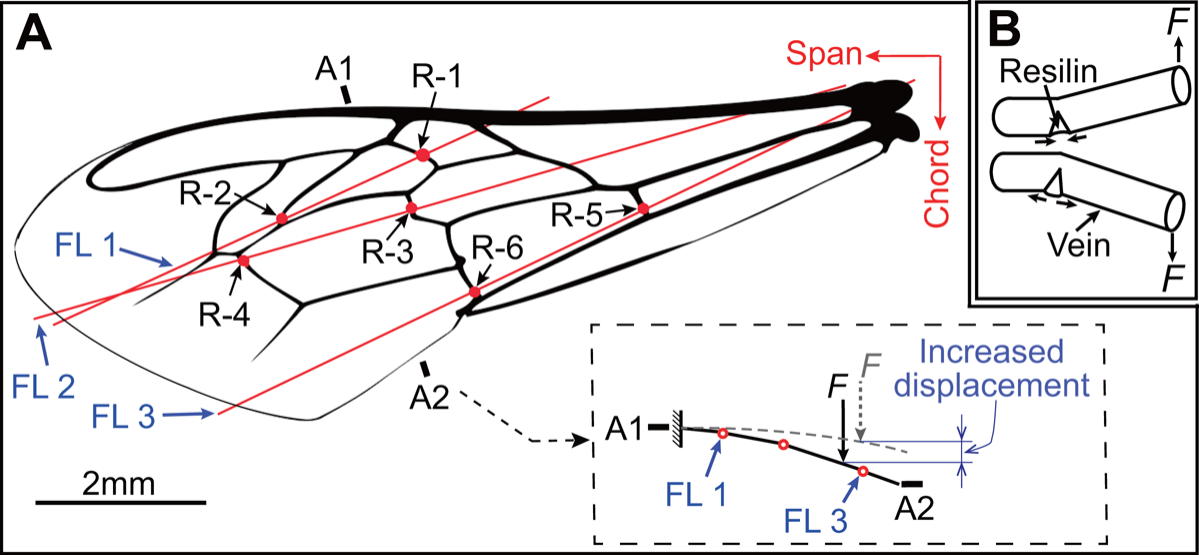
**Illustration of chordwise flexibility of the honeybee forewing.** (A) The function of resilin distribution on the chordwise bending. R-1–R-6 represent the six resilin patches in the forewing. FL refers to flexion lines. (B) Schematic diagram of the function of resilin in veins.

The fluorescence microscopy of the forewing identified six resilin patches on the ventral side of the forewing (Fig. 3). All the six resilin patches were embedded in cross-veins along the chord nearer to vein-joints or longitudinal veins. This caused a reduction of the structural integrity of the cross veins and allowed them to flex more easily (Fig. 4) owing to the rubber-like high resilience of resilin (Lv et al., 2010; Weis-Fogh, 1960). Based on the distribution of resilin patches, the relative positions of forewing veins and the observed wing deformations during flight (Ma et al., 2015), three flexion lines can be hypothesized to exist in the forewing that are conducive to understanding the wing deformation. As indicated in Fig. 4A, the flexion lines are axes of the wing profile deformation during flapping flight, which most likely increase the chordwise flexibility. This will make the wing return to its initial position rapidly after the elastic deformation caused by the deformed resilin patches when no external forces are acting on the wing (Gorb, 1999; Donoughe et al., 2011). Consequently, the flexion lines potentially facilitate the chordwise bending of the forewing compared with the wing without resilin patches (grey dot-curves in Fig. 4A). Thus, this would allow the forewing to bend more easily along the chord than along the span, and have a strong influence on the span-chordwise bending anisotropy of the forewing.

### Micro-CT scanning

**Fig. 5.**
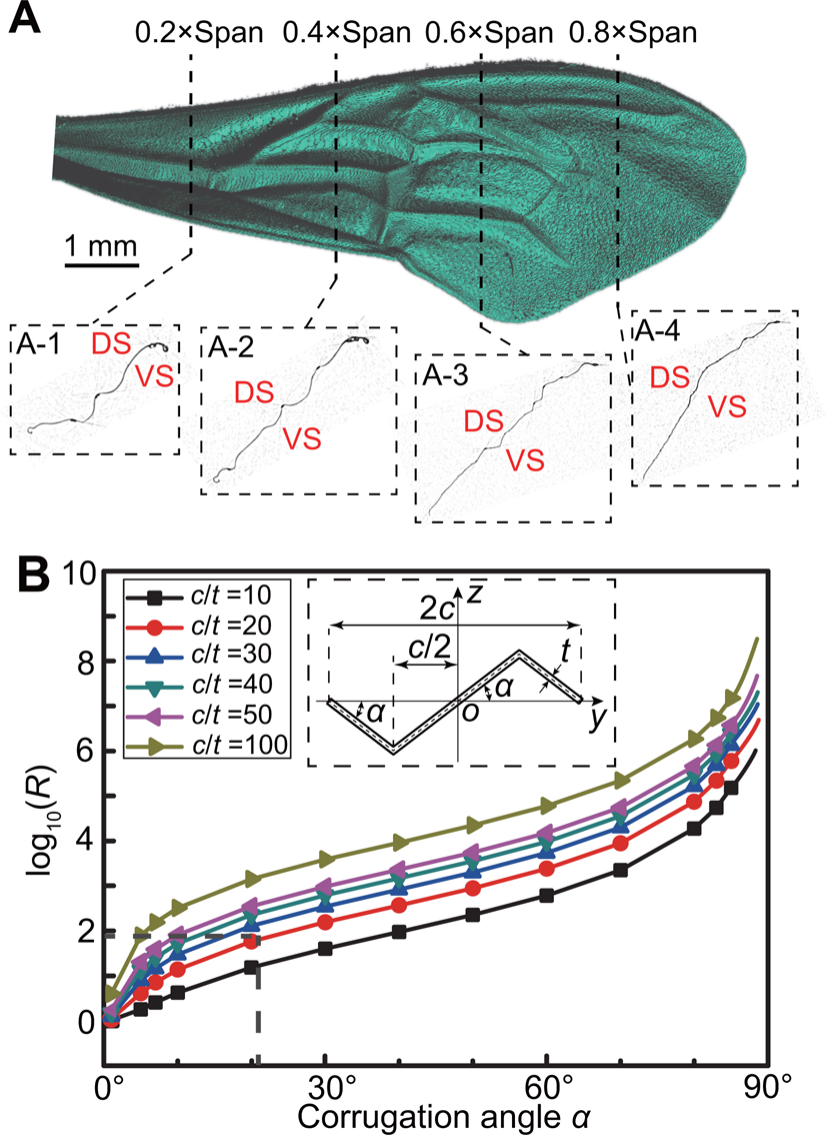
**Illustration of wing corrugation along the span.** (A) Reconstructed micro-CT wing (from dorsal side) and inherent cross-sections of the forewing along various spanwise positions (A-1–A-4). DS and VS refer to the dorsal and ventral sides, respectively. (B) Cross-section of a corrugation element and ratio between the bending stiffness of corrugated and rectangular elements versus corrugation angle *α* with different ratio of chordwise length of the corrugated cross-section and membrane thickness. *α* is corrugation angle, 2*c* is the chordwise length of a single corrugated element, and *t* is membrane thickness. The vertical axis is on a logarithmic scale.

**Fig. 6.**
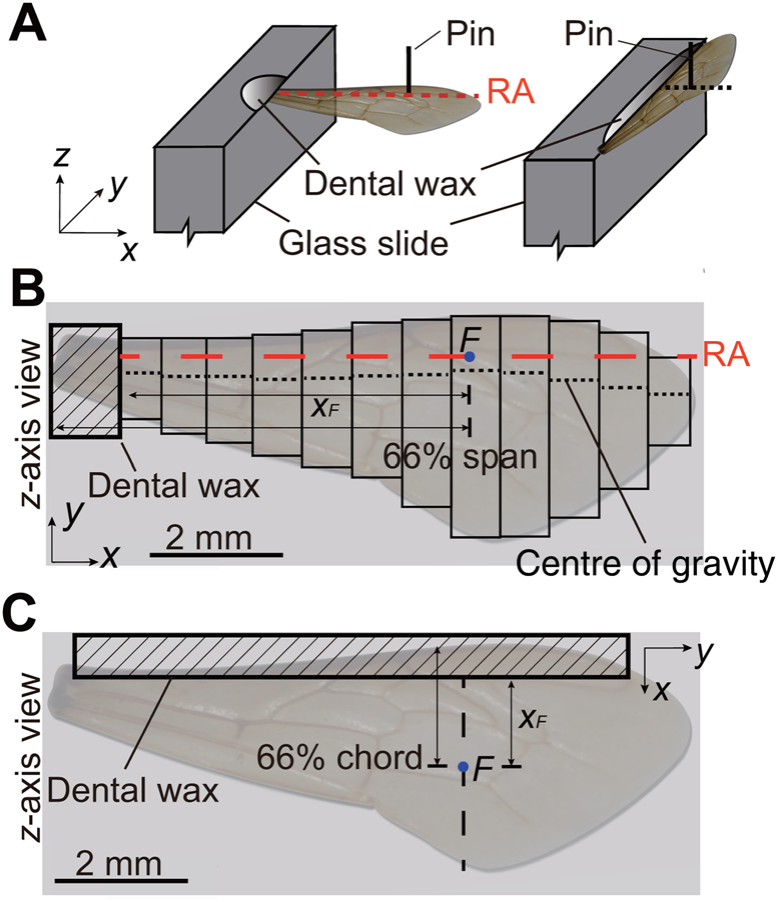
**Illustration of the forewing immobilization.** (A) The forewing is fixed to the glass slide using dental wax. As indicated by Sunada et al. (1998) and Ganguli et al. (2010), a needle is used to apply a force at different spanwise positions and the points where only bending resulted from the applied force could be connected to determine the rotational axis (RA), which is closer to the leading edge of the wing than the gravity centre. (B) The *z*-axis view of the loading circumstance and specimen immobilization. The wing can be viewed as a stepped cantilevered beam with varied width, generating multiple chordwise strips. *x_F_* is the effective length of bending. (C) The *z*-axis view of the chordwise loading circumstances and specimen immobilization.

After longitudinally scanning the forewing with the micro-CT scanner, four chordwise cross-sections of four spanwise positions, i.e. 0.2×span, 0.4×span, 0.6×span, and 0.8×span, were obtained as shown in Fig. 5A. These clearly show the cross-sectional corrugation of the forewing, especially at the wing base and proximal parts. The spatial layout of longitudinal supporting veins in the spanwise direction (Fig. 5A) induces relatively longer ‘ridges and valleys’ (Ma et al., 2015, 2017) on the wing surface, causing the longer and narrower corrugation along the span. However, the shorter cross-veins principally connect the longitudinal veins along the chord, and could not generate the longer and narrower corrugation along the chord (Wootton, 1981). Hence, functional aspects of the chordwise and spanwise sections can be approximately viewed as the corrugated and rectangular sections, respectively. However, according to the force position, the width of the chordwise section (Fig. 6B) is nearly twice that of the spanwise section (Fig. 6C). We could therefore evaluate the ratio *R* between *EI* of a corrugated section (Fig. 5B) and *EI* of rectangular section as follows, assuming that they have the same Young's modulus *E* using:
(3)
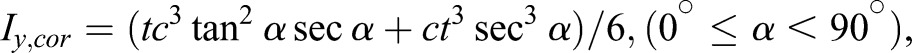

(4)
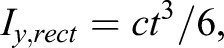
 and
(5)
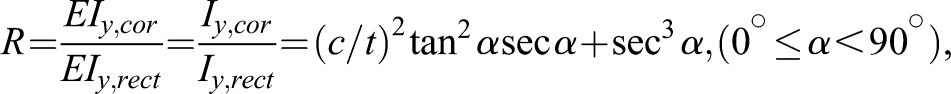



where *I_y,cor_* is the cross-sectional inertia moment of the corrugated element (Fig. 5B), *I_y,rect_* is the cross-sectional inertia moment of the rectangular element, and the other symbols are as defined in Fig. 5B. The ratio *R* is proportional to the second power of *c/t* and increases with the wing becoming thinner or more corrugated (Fig. 5B).

The averaged corrugation angle *α* and ratio *c/t* of the chordwise length and membrane thickness of insect wings were estimated to be 10–40° and 15–40°, respectively (Rees, 1975a). In our study, according to the force positions during the bending test (Fig. 6B), the cross-section at position 0.6×span (Fig. 5A-3) can be chosen as the calculated cross-section, in which the averaged chordwise length of the corrugation element can be determined as 0.6 mm. Considering the averaged membrane thickness, 10 μm, and averaged corrugation angle, 21.1°, *c/t* can be calculated as 30; thus, the ratio *R* indicates that *EI_y,cor_* is nearly two orders of magnitude larger than *EI_y,rect_* (Fig. 5B). In summary, the corrugation obviously increases the second moment of area of the forewing section, and the values of *c/t* and *α* together determine the relatively greater spanwise *EI_y,cor_* of the corrugated honeybee forewing. To some extent, this agrees well with the result of the bending test that the spanwise *EI* is two orders of magnitude larger than the chordwise *EI*; however, this is just a theoretical analysis made with some assumptions, wing corrugation could be regarded as one of several major factors and is potentially not the dominant factor determining the span-chordwise bending anisotropy.

For corrugated dragonfly wings, the corrugation patterns and leading edge orientation are different along the span (Lian et al., 2014), and this kind of conformation typically allows supinatory twisting while restricting pronatory twisting and permits the passive upstroke torsion (Wootton et al., 1998). In the gliding performance of dragonflies, their corrugated wings perform best with a lift-to-drag ratio higher than that of flat wings. Meanwhile, corrugated wings attain higher lift values and smaller drag values than flat wings (Kesel, 2000; Chen and Skote, 2016). However, in their comparison with Kesel's research (Kesel, 2000), Chen and Skote (2016) pointed out that the variation of leading edge orientation along the wing span is the crucial detail for preventing oscillations of lift and drag. In addition, strong spanwise flow occurs in the 3D corrugated wing used in their study, which could not be captured by previous models. Thus, with a Reynolds number of a few hundred, it seems that this wing corrugation has all the advantages of low mass, high stiffness, and low membrane stresses in bending associated with corrugation, but without any obvious aerodynamic shortcomings, as compared with the smooth or flat profile (Rees, 1975b).

### Wing venation

In an earlier finite element method (FEM) study of the *Manduca* wing (Combes and Daniel, 2003), it was verified that leading edge veins, the supporting longitudinal veins with larger diameter, cause the span-chordwise anisotropy of the wing. It was demonstrated that an FEM model of the wing without any supporting leading edge veins would lead to similar spanwise and chordwise *EI*; while the model with leading edge veins had dramatically increased spanwise *EI*, generating the span-chordwise anisotropy. Hence, considering that the leading edge vein is a common venation feature among insect wings (Chen et al., 2013; Kesel et al., 1998), and also appears in the honeybee forewing (Fig. 4A), the span-chordwise bending anisotropy of the forewing could be partly attributed to the leading edge vein. This supporting longitudinal vein raises the spanwise *EI* of the forewing; nevertheless, no obvious changes occur on the chordwise *EI*.

### Significance of the span-chordwise bending anisotropy

From a structural perspective, this span-chordwise anisotropy would serve to control wing shape changes. It would strengthen the forewing from bending along the wing span, but also permit the chordwise bending to camber the wing, namely the typical ‘umbrella effect’ (Wootton, 1995). Thus, it would further promote the torsion along the wing span, which has been confirmed by the observation of supination and pronation (Ma et al., 2015; Wootton, 1981; Ennos, 1988a,b; Walker et al., 2010) of many insects in free flight, especially contributing to the indispensable transition for stroke reversals between upstrokes and downstrokes (Ma et al., 2015).

Moreover, the aerodynamic performance could be enhanced by the chordwise flexibility and spanwise stiffness of the wing. The flexibility along the chord is conducive to reinforcing load-lifting capacity, power efficiency, and wing propulsion efficiency (Zhu, 2007; Vanella et al., 2009; Mountcastle and Combes, 2013; Liu et al., 2013). In addition, the camber effect may regulate the magnitude of the lift and drag ratio and control the alteration of aerodynamic forces (Walker et al., 2009; Zhao et al., 2010). However, the wing is mainly supported by the corrugated longitudinal veins along the span, particularly the leading edge veins. In this case, the resultant spanwise stiffness restricts the spanwise bending deformation of the leading edge in order to, we think, stabilize the strong leading-edge vortex and the high axial flow to achieve high lift production during hovering and forward flight (Ellington et al., 1996; Berg and Ellington, 1997a,b). In brief, the span-chordwise bending anisotropy is closely correlated with the structural and aerodynamic characteristics of insect wings.

### Conclusion

In conclusion, the spanwise and chordwise bending stiffness *EI* of the honeybee forewing were evaluated using a cantilevered bending test. It was found that the spanwise *EI* is nearly two orders of magnitude larger than the chordwise *EI*. This span-chordwise anisotropy is mainly attributed to three factors, namely distribution of resilin patches, wing corrugation along the wing span, and wing venation. Flexion lines formed by the resilin patches potentially facilitate the chordwise bending of the forewing during flapping flight. Moreover, the wing corrugation and leading edge veins of the venation both determine the relatively greater spanwise *EI* of the corrugated wing.

This anisotropy significantly endows the insect wings with specific structural and aerodynamic features. On one hand, wings could be bent more easily along the chord than along the span, beneficial for generating the ‘umbrella effect’, spanwise torsion, and stroke reversals. On the other hand, this anisotropy is capable of enhancing the aerodynamic performance, especially producing high lift during hovering and forward flight. The novel concepts of the present work may provide some inspirations for the engineering of bio-inspired wings for flapping micro-aerial vehicles.

## MATERIALS AND METHODS

### Specimen preparation

Worker honeybees (*Apis mellifera*) were obtained from the Bee Research Institute, Chinese Academy of Agricultural Sciences, Beijing, China, and raised in a humidified container at room temperature (approximately 20°C). Each bending test of the fresh wing was conducted within 10 min (Smith et al., 2000), whenever possible, of removing the wings from the honeybee; this was intended to prevent changes to the mechanical properties of the wing that might be caused by desiccation.

### Bending test

The smaller size of the hindwing caused difficulties performing tests; thus, this study only focuses on the forewing of the honeybee. The wing base was immobilized at one end of the glass slide along its thickest direction using dental wax (Fig. 6A-C), and the other end of the glass slide was fixed at the clamp of the material testing system (MTS) (Tytron 250, MTS Systems Corporation, USA), whose load range is 0.001 N to 250 N, minimum displacement is 0.001 mm, and frequency is 50 Hz. The test duration and moving distance of loading pin were respectively set to be 300 s and 1.5 mm. The pin was fixed at the other clamp to provide the test load; the blunt end of the pin was used as the loading end to avoid impaling the wing. Four types of tests of the forewing were performed, aimed at measuring the spanwise bending stiffness when bending down and up and the chordwise bending stiffness when bending down and up, respectively. The maximum spanwise and chordwise lengths of the forewing were ∼9.3 mm and ∼3.3 mm, respectively.

### Fluorescence microscopy

To investigate the resilin distribution of the forewing, two forewings (dorsal side and ventral side up) were observed in a fluorescence microscope (Leica DMI 6000B, Leica Microsystems, Germany) using UV bands (excitation 340-380 nm, emission 425 nm). Wings were removed from the anesthetized honeybee, and then were cleaned using alcohol to remove as much dust as possible and improve the sharpness of the images. However, resilin in biological structures shows autofluorescence only in a very narrow wavelength band of approximately 400 nm (Andersen and Weis-Fogh, 1964). The use of UV bands meant that there was no need to use immune labelling and other treatments to reveal the autofluorescence of resilin, and thus maintained the specimens in as natural a state as possible. Lab temperature and humidity were maintained at 25°C and 60%.

### Micro-CT

A SkyScan 1172 micro-CT scanner (Bruker microCT, Kontich, Belgium) was used to create high-resolution cross-sectional images of the wing. The forewing was scanned with a resolution of 4.05 μm at 40 kV and 250 μA, which means that pixels in the resulting cross-sectional images correspond to a dimension of 4.05×4.05 μm^2^. The total scan consisted of 2098 cross-sectional images of the forewing, and the 3D rendering was conducted using CT-vox software (version 1.5, Bruker microCT, Kontich, Belgium). The extremely thin forewing meant that the degree of colour contrast and virtual lighting had to be adjusted to achieve clear rendering (Fig. 5A).
